# Comparative Mitogenomic Analysis of Heptageniid Mayflies (Insecta: Ephemeroptera): Conserved Intergenic Spacer and tRNA Gene Duplication

**DOI:** 10.3390/insects12020170

**Published:** 2021-02-16

**Authors:** Ran Li, Zhiming Lei, Wenjuan Li, Wei Zhang, Changfa Zhou

**Affiliations:** The Key Laboratory of Jiangsu Biodiversity and Biotechnology, College of Life Sciences, Nanjing Normal University, Nanjing 210023, China; 90716@njnu.edu.cn (R.L.); 201202011@njnu.edu.cn (Z.L.); 201202051@njnu.edu.cn (W.L.); 171201004@njnu.edu.cn (W.Z.)

**Keywords:** Ephemeroptera, Heptageniidae, mitochondrial genome, intergenic spacer, gene rearrangement

## Abstract

**Simple Summary:**

Heptageniidae is one of the most abundant and widespread families of mayflies, with more than 600 described species and distributed mainly in the Holoarctic, Oriental, and Afrotropical regions. Previous phylogenetic analyses in this family mainly focused on morphological characters and were restricted to one or several genes. Ten complete mitochondrial genomes from this family are reported here. A conserved intergenic spacer and tRNA gene duplication in Heptageniidae mitogenomes may be regarded as ancient features and provide evidence for the phylogenic analysis within Ephemeroptera.

**Abstract:**

Large intergenic spacers and tRNA gene duplications have been reported in several insect groups, although little is known about mitogenomes of mayflies. Here, we determined complete mitogenomes of ten heptageniid species and systemically analyzed their mitogenomic features. Both a conserved intergenic spacer (IGS) and *trnM* duplication were detected in those mitogenomes. The IGS, which was observed in heptageniids, could be further folded into a stable stem–loop structure. The tRNA gene duplication was found in almost all analyzed mitogenomes, and a unique gene block *trnI*-*trnM*-*trnQ*-*trnM*-*ND2* was also discovered. Our analysis demonstrates that the heptageniid gene arrangement pattern can be explained by the tandem duplication-random loss (TDRL) model. Phylogenetic analyses using both Bayesian inference (BI) and maximum likelihood (ML) methods based on the nucleotide and amino acid sequence data recovered the genus *Epeorus* as monophyletic with strong support. Our results provide a better understanding of mitogenomic evolution in Heptageniidae, as well as novel molecular markers for species identification of mayflies.

## 1. Introduction

The mitochondrial genome (mitogenome) is a double-stranded DNA molecule with 14−20 kb long and accounts for 1–2% of the total genome found in cells [1,2]. A complete insect mitogenome generally consists of 37 genes, specifying 13 protein-coding genes (PCGs), 22 transfer RNAs (tRNAs), 2 ribosomal RNAs (rRNAs), and a non-coding control region (CR) [3,4]. The sequence of insect mitogenome is a commonly suitable molecular marker for phylogenetic and evolutionary studies, due to its maternal inheritance, high nucleotide substitution rate, simple genetic structure, and lack of recombination [2,5,6,7]. Dramatic variation in gene organization was found in some insect groups. The gene rearrangements and/or intergenic spacer (IGS) regions appear to be regarded as an additional informative character for phylogenetic reconstruction among some groups [8,9,10].

Mayflies (Ephemeroptera) are one of the most archaic extant winged insect lineages. The amphibiotic insects with highly reduced terrestrial adult stage have evolved considerably for about 300 Mya [11]. Compared with many other insect orders, the available data of mitogenome from Ephemeroptera are quite scarce. There are currently only 38 complete or partial mitogenomes (among 12 families) deposited to the GenBank (https://www.ncbi.nlm.nih.gov/; 20 December 2020). The relative positions of PCGs and rRNAs are usually stable and conserved among different ephemeropteran species, however, tRNAs display different degrees of variation. In the mitogenome of *Siphluriscus chinensis* (Siphluriscidae), for example, *trnK* is duplicated and translocated at the upstream of *trnE*, which generates a new gene cluster (*trnS1*-*trnK*-*trnE*) compared with the putative ancestral gene order [12]. The rearrangement of several tRNAs is found in *Alainites yixiani* (Baetidae) with a gene cluster of *trnI*-*CR*-*trnC*-*trnQ*-*trnY*-*trnM*-*ND2*-*trnW* [13]. Recent studies have also demonstrated that *trnI* of Ephemerellidae is inverted from the downstream of CR in the H-strand to the upstream of CR in the J-strand, forming an identical gene block (*trnI*-*CR*-*trnQ*-*trnM*) [14]. In addition, the duplication of *trnM* gene is always observed in Heptageniidae, except *Paegniodes cupulatus* [13,15,16]. It is apparent that more mitogenomes from diverse groups of mayflies are in demand to well understand the mechanism of these gene rearrangements in the following studies.

Heptageniidae is a family with abundant species-diversity, and more than 600 species belonging to three subfamilies (Ecdyonurinae, Heptageniinae and Rhithrogeninae) have been described [17]. The species are widely distributed in the Palaearctic Region, and their nymphs generally inhabit the surface of rocks, leaves and vegetations in distinct kinds of lotic freshwater habitats (rivers, lakes and streams) [18]. Because of their sensitivity to pollution, many species have been used as excellent models for aquatic biological monitoring and biodiversity researches [19,20]. Over the past decade, the taxonomic studies of this family are mainly based on morphological characters of different life stages [17,21,22,23]. However, it remains a challenge to identify the heptageniid mayflies because of a lack of dependable characters for both two major stages (nymph and adult) [19]. Consequently, it is generally not possible to obtain definite species names in the research in which immature individuals of the sample account for a large proportion [20,24]. Furthermore, phylogenetic relationships among different groups in this family are still controversial with limited molecular information [25,26]. Only six reliable mitogenome sequences have previously been published for Heptageniidae to date, with four mitogenomes belonging to the genus *Epeorus*, one mitogenome of the genus *Parafronurus*, and one of the genus *Paegniodes*. At the same time, most previous related studies tended to focus on one species and its phylogenetic placement in Ephemeroptera [13,15,16].

Accordingly, we used next-generation sequencing method to obtain mitogenomes for 10 heptageniid mayflies, which represented all three subfamilies: five species from Rhithrogeninae, four from Ecdyonurinae, one from Heptageniinae. We compared the newly generated mitogenomes to the six previously reported sequences, described the structural and compositional features of heptageniid mitogenomes and analyzed the intergenic spacers to access possible evolutionary mechanisms. We also analyzed gene rearrangement patterns and discussed possible rearrangement processes and mechanisms in detail. Finally, the phylogeny of Heptageniidae was reconstructed combining all available mitogenomes.

## 2. Materials and Methods

### 2.1. Sample Collection and DNA Extraction

Ten specimens of Heptageniidae were collected between 2019 and 2020 from different sampling sites in China. Detailed information is shown in Supplementary Materials Table S1. Morphological identification of the specimens was conducted by C.F. Zhou based on the key diagnostic features. All samples were deposited in 100% ethanol at −20 °C and then cataloged in the voucher collection of Mayfly Museum of Nanjing Normal University, Jiangsu Province, China. Total genomic DNA extraction was performed from the leg tissue using TIANamp Genomic DNA Kit (TIANGEN, China) according to the manufacturer’s protocol. DNA quality and concentration were measured on the Nanodrop 2000 spectrophotometer and visualized on 1.0% agarose gel. The qualified DNA was preserved at −20 °C and used for sequencing.

### 2.2. Mitogenome Sequencing and Assembly

Genomic DNA of all samples was sent to Personalbio Inc. (Shanghai, China) for library construction and next-generation sequencing (NGS). One library (Insert size of 400 bp) was prepared for each DNA sample using the TruSeqTM DNA Sample Prep Kit (Illumina, USA). All constructed libraries were then sequenced as 150 bp paired-end on a full run (2 × 150 PE) using the Illumina NovaSeq platform. After trimming the adapter contamination and removing short and low-quality reads, more than 4 GB (30−41 million reads) clean data for each sample was used in de novo assembly (Table S2). The complete circular mitogenomes were assembled using NovoPlasty 4.0 (k-mer = 33), with the *COI* gene of *S. chinensis* as a seed sequence (Accession number: HQ875717).

### 2.3. Gene Annotation and Bioinformatic Analysis

All newly determined mitogenomes were firstly annotated using MitoZ 2.4 pipeline [27]. The parameter setting for Genetic Code 5 (invertebrate) was selected, and the Arthropoda database was used to select reference sequences. Secondary structure of tRNAs was predicted by tRNA scan-SE 2.0.2 and ARWEN 1.2 to confirm their accuracy [28,29]. The boundaries of two rRNAs were identified using the ClustalW algorithm in MEGA 7.0 based on alignments of other available heptageniid mitogenomes [30]. PCGs with non-canonical start and stop codons were further adjusted and corrected manually by translating into amino acids.

The nucleotide composition of all components and the relative synonymous codon usage (RSCU) of PCGs were estimated using MEGA 7.0. The base composition values (AT- and GC-skews) were calculated using the following formulas: AT-skew = (A − T)/(A + T) and GC-skew = (G − C)/(G + C) [31]. The sliding window analysis (a sliding window of 200 bp and step size of 20 bp), and the nucleotide diversity (Pi) of 13 PCGs among 16 mitogenomes of Heptageniidae were conducted using DnaSP 6.0 [32]. The numbers of synonymous substitutions (Ks) and non-synonymous substitutions (Ka), and the ratios of Ka/Ks for each PCG were also measured in the software DnaSP 6.0. The genetic distances based on Kimura-2-parameter among the 16 mitogenomes were analyzed by MEGA 7.0.

### 2.4. Phylogenetic Analysis

A total of 17 mitogenomes of Ephemeroptera were used for the phylogenetic analyses, including 16 species of Heptageniidae (10 newly sequenced mitogenomes and six downloaded from the GenBank) as the ingroups and one species of Siphluriscidae (*S. chinensis*) as outgroup (Table 1). Phylogenetic analyses were reconstructed on the concatenated datasets of 13 PCGs at both amino acid and nucleotide levels with Maximum likelihood (ML) and Bayesian analysis (BI) methods. Individual PCG sequences from all the 17 species (excluding the start and stop codons) were aligned individually with codon-based multiple alignments using the software MUSCLE 3.8.31 [33]. The individual aligned sequences were then concatenated by PhyloSuite [34], and conserved regions were identified by the program Gblock 0.91b [35]. The optimal partitioning strategy for two datasets was inferred using PartitionFinder 2 in the PhyloSuite program [36], under a greedy search algorithm with linked branch lengths based on the Bayesian information criterion (BIC) (Tables S3 and S4). Bayesian phylogenetic analyses were conducted using MrBayes 3.2.6 with default settings as implemented in the CIPRES [37,38], and ran for 10^6^ generations sampling every 1000 generations. The first 25% of generations were removed as burn-in, and the average standard deviation of split frequencies < 0.01 was considered to reach convergence. ML analyses were carried out by RAxML 8.2.0 with a GTRGAMMAI model. The branch support for each node was evaluated with 1000 bootstrap replicates [39].

## 3. Results and Discussion

### 3.1. Genomic Organization and Composition

**Figure 1:**
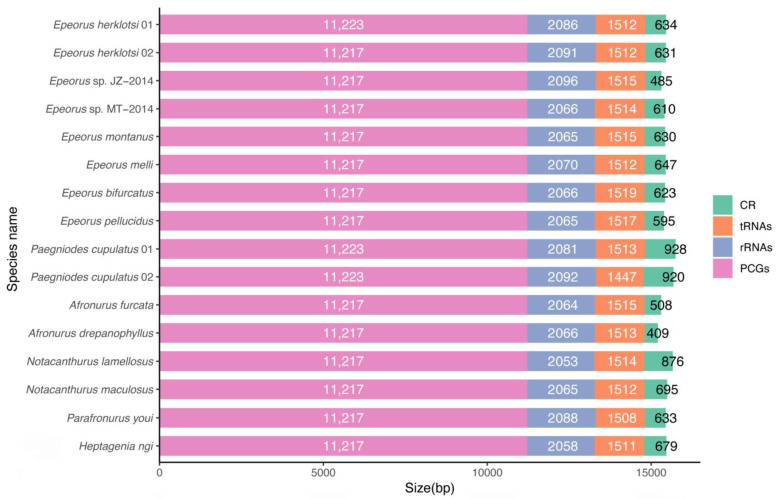
The length of protein-coding genes (PCGs), transfer RNAs (tRNAs), ribosomal RNAs (rRNAs), and control regions (CRs) among 16 heptageniid mitogenomes.

**Figure 2:**
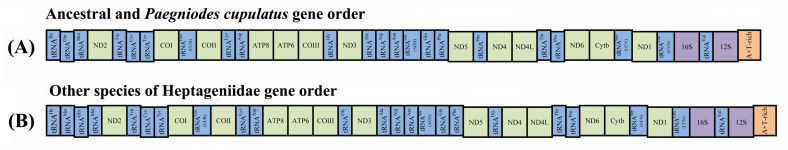
(**A**) The ancestral and *Paegniodes cupulatus* gene order. (**B**) The gene order of other heptageniid mitogenomes.

We successfully obtained the complete mitogenomes of ten mayflies of the family Heptageniidae. Sequences were deposited in GenBank with the accession numbers: MW381291−MW381300. The total length of all new mitogenomes was well within the range of known complete mitogenomes in Ephemeroptera, with sizes from 14,589 bp in *A. yixiani* to 16,616 bp in *S. chinensis* [12,13]. The lengths of PCGs, tRNAs, rRNAs, and control regions (CRs) for the ten mitogenomes and other six reported mitogenomes of Heptageniidae are compared in Figure 1. Size variation of mitogenomes was mainly due to the difference in the size of the CRs. Nine of the newly determined mitogenomes also contained 38 genes: 13 PCGs, 23 tRNAs, and 2 rRNAs (Figure 2), and an additional *trnM* gene was identified in most Heptageniidae. The mitogenome of *P. cupulatus* encodes the typical 37 genes, which is consistent with a previously reported sequence. The accuracy of the existing mitogenome of *P. cupulatus* sequenced using Sanger technology was verified by our new generated sequence [16]. Most of the genes (9 PCGs and 15 (14) tRNAs) were encoded on the major strand (J-strand), while the remaining genes were on the minor strand (N-strand) (Figure 2).

As is typical for insects, the analyzed mitogenomes had a biased nucleotide composition with A + T content ranging from 63.68% (*Afronurus drepanophyllus*) to 66.77% (*Notacanthurus maculosus*) (Table 2). For most species, the highest A + T content was found in CRs (11 species), and only six species showed the highest A + T content in rRNAs. The relative numbers of A to T and G to C were measured by AT-skew and GC-skew of the base composition in nucleotide sequences. The results of skewness statistics showed that the AT-skews were slightly negative (−0.002 to −0.046) in complete mitogenomes among heptageniids, while GC-skews were obviously negative (−0.176 to −0.245). Meanwhile, PCGs of all mitogenomes also had a negative AT-skew and GC-skew, which indicated that T and C content is significantly more abundant than A and G.

### 3.2. Protein-Coding Genes

**Figure 3:**
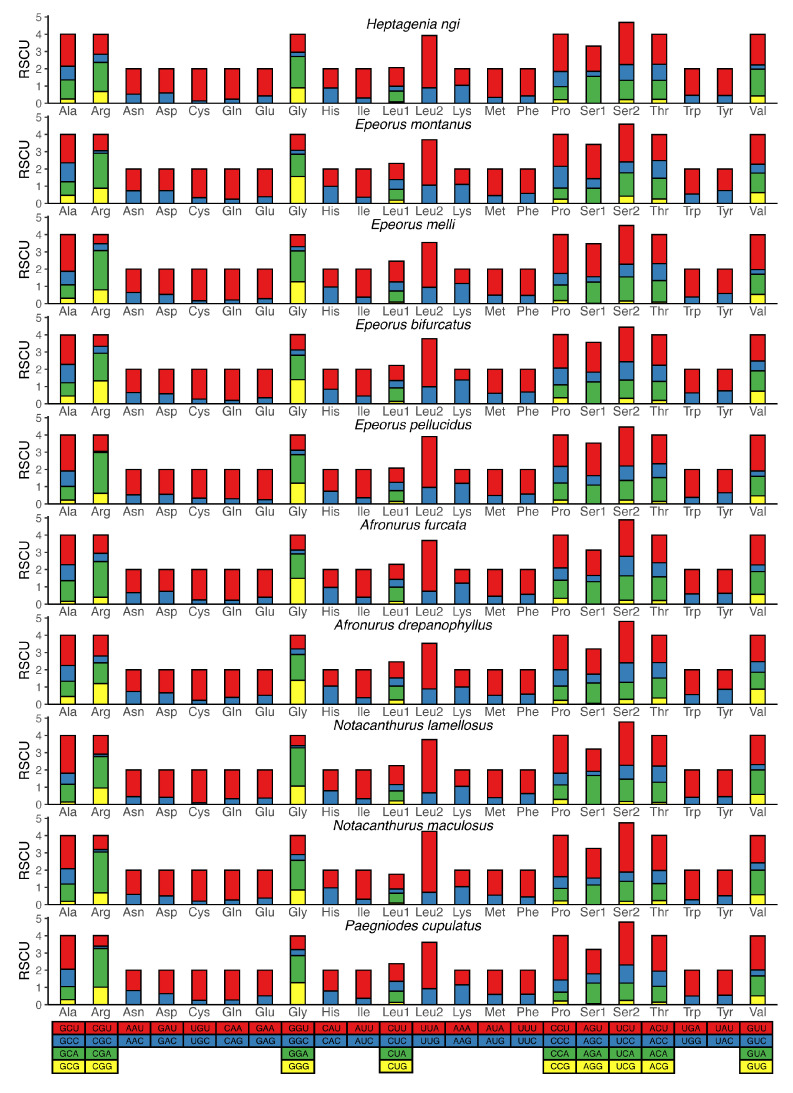
Relative synonymous codon usage (RSCU) of ten heptageniid mitogenomes. Codon families are provided on the X-axis along with the different combinations of synonymous codons that code for that amino acid. RSCU is defined on the Y-axis.

All 13 PCGs were detected in the newly generated mitogenomes and their general characters were similar with the total length of 11,217 bp and 11,223 bp (Figure 1). The orientations of the PCGs were identical to other available mitogenomes of mayflies: four genes (*ND1*, *ND4*, *ND4L* and *ND5*) were encoded on the N-strand, and the remaining nine were on the J-strand. The majority of PCGs used the typical start codons ATN (ATA, ATG, ATT and ATC) with the exception of *COI* and *ND5* in most species, which used ACC and GTG (Table S5). Most PCGs terminated with the conventional TAA and TAG as stop codons while three genes (*COII*, *ND4* and *ND5*) terminated as truncated T. Incomplete terminating codons are a common phenomenon, related to post-transcriptional modification during mRNA maturation [40,41]. As shown in Figure 3, the relative synonymous codon usage (RSCU) was generally similar with each other in newly sequenced mitogenomes. Overall codon usage analysis showed that the codons ending with A or T were more preferred. The five most frequently used codons were UUA (Leu2), UCU (Ser2), CCU (Pro), GCU (Ala) and UGU (Cys), and the highest value of RSCU was 3.54 in *N. maculosus* (Figure 3). Our results reveal that the size, orientation and RSCU of PCGs are relatively conserved among the Heptageniidae.

**Figure 4:**
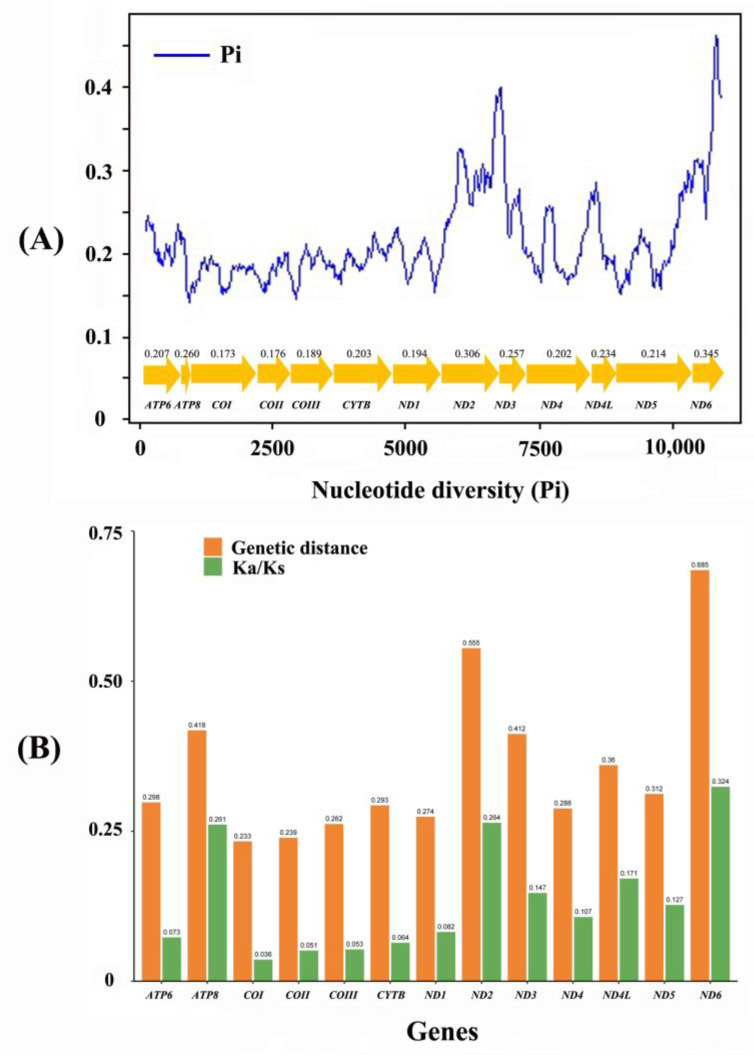
(**A**) Sliding window analysis based on 13 aligned PCGs. The blue line shows the value of nucleotide diversity Pi. The gene names and Pi values are shown in the graph. (**B**) Genetic distance (on average) and non-synonymous (Ka) to synonymous (Ks) substitution rates of 13 PCGs among 16 heptageniids.

Nucleotide diversity was analyzed with a sliding window of the 13 aligned PCGs (Figure 4A). *ND6* (Pi = 0.345) and *ND2* (Pi = 0.306) had apparently higher nucleotide diversity than other genes, while *COI* had the lowest Pi of 0.173. Analysis of pairwise genetic distance showed similar results with *ND6* (0.685) and *ND2* (0.555) evolving relatively faster, while *COI* (0.233) and *COII* (0.239) were slower (Figure 4B). Average Ka/Ks ratios were estimated to investigate evolutionary rates of mitogenome PCGs. Ratios ranged from 0.036 for *COI* to 0.324 for *ND6*, which indicated that all PCGs were under purifying selection. Our results showed *ND6* and *ND2* exhibited relaxed purifying selection, while *COI* was under the strongest purifying selection (Figure 4B).

Analyses of nucleotide diversity, genetic distance and evolutionary rate are useful for designing specific markers among different groups, especially in the taxa with highly variable morphological characters. Our comprehensive analysis showed that *COI* had the lowest evolution rate and evolves under comparative relaxed purifying selection, two genes (*ND6* and *ND2*) exhibited faster evolution rate and diversity than other PCGs, which is inconsistent with previous studies that *COI* was usually considered as one useful marker for species identification and phylogenetic analysis in closely-related taxa [42,43]. Overall, *ND6* and *ND2* might be two potential markers for identifying cryptic species, reconstructing phylogenetic trees and phylogeographic analysis in Heptageniidae.

### 3.3. Ribosomal and Transfer RNAs

Both rRNAs (*rrnL* and *rrnS*) were found in the mitogenomes of all ten heptageniid mayflies, encoded on the N-strand and located between the *trnL1* and *trnV*, and between *trnV* and the CR, respectively. Among all analyzed mitogenomes of Heptageniidae, *rrnL* length ranged from 1278 bp in *P. cupulatus* to 1291 bp in *Epeorus* sp. JZ 2014 (Figure 1). The longest *rrnS* was shown in two sequences of *P. cupulatus* and the shortest was in *Notacanthurus lamellosus*. A + T content of the rRNAs ranged from 63.95% to 67.36%, which exhibited a high A + T bias (Table 2). All 22 typical tRNAs found in insect mitogenomes were observed in the newly sequenced mitogenome of *P. cupulatus*, and an additional *trnM* was also found in the remaining nine mitogenomes. The length of all tRNAs ranged from 61 to 71 bp and could be folded into a typical clover-leaf secondary structure with the exception of *trnS1* due to a lack of the DHU arm. Loss of the DHU arm in *trnS1* is a common feature in insect mitogenomes [44].

### 3.4. Non-Coding Regions

Different numbers and sizes of non-coding regions are observed in metazoan mitogenomes, which results in variable size mitogenome between species [45]. Of the non-coding regions, the largest one is usually thought to be the control region, which contains signals for regulation and initiation of mitochondrial DNA transcription and replication [46,47]. The CRs of heptageniid mitogenomes were located in the conserved position between *rrnS* and *trnI* genes (Figure 2). The length of *P. cupulatus* 02 was longer than that of other species, resulting in having the longest mitogenome (Figure 1). There were large length variations across species, even closely related ones, which supports a previous study [48]. Rapid variation of CRs seemed to provide information for species evolution, but its internal mechanisms need deeper examination.

**Figure 5:**
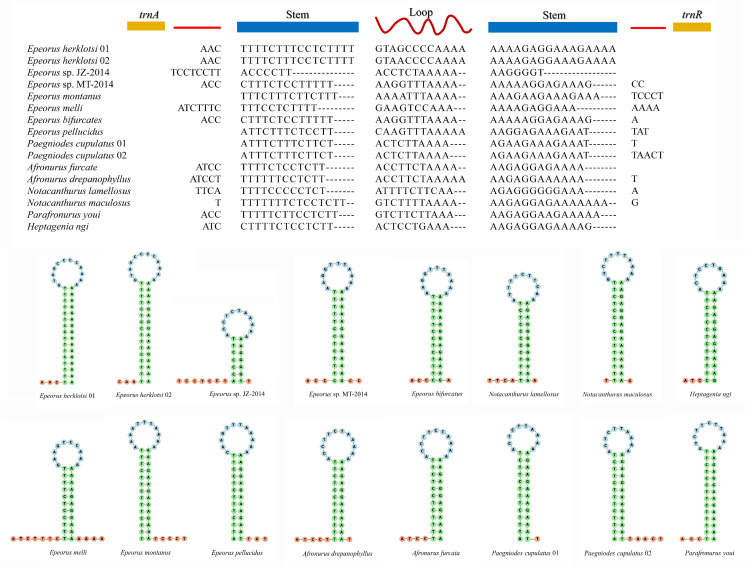
Alignment of the conserved intergenic spacer (IGS) between *trnA* and *trnR* of heptageniid mitogenomes and the putative stem–loop structures.

The most remarkable genomic feature was the presence of one intergenic spacer (IGS) region between *trnA* and *trnR* in all heptageniid mitogenomes. The IGSs of different species had different lengths (32–47 bp) and base compositions, but had a similar secondary structure. As shown in Figure 5, all IGSs could be folded into a stable stem–loop structure. The features mentioned above (stem–loop hairpin structure, a size of approximately 30 nucleotides, and a stem with complementary bases) were similar to the mitochondrial origins for replication of L-strand (OL) that existed in vertebrate mitogenomes [49,50]. This OL-like region is only observed in heptageniids, but not found in other families of Ephemeroptera. We hence assumed that the conserved region is a molecular synapomorphy of the family Heptageniidae. This region is commonly found in vertebrate mitogenomes, however, it has not been reported in insect mitogenomes so far [51,52].

### 3.5. Gene Arrangement

Across the order Ephemeroptera, several gene rearrangement events have been observed, such as in *S. chinensis*, *A. yixiani* and *C. fusca* [12,13,14]. Comparative analysis of the heptageniid mitogenomes indicates that the *trnM* duplication is unique to the family. The ancestral gene composition (22 tRNAs), however, is still recorded in the mitogenome of *P. cupulatus*. Interestingly, only this species shows the ancestral arrangement pattern. However, whether this event was identical with another species of this genus of *Paegniodes* (*P. dao*) remains unclear, due to the lack of sequence information for that group.

**Figure 6:**
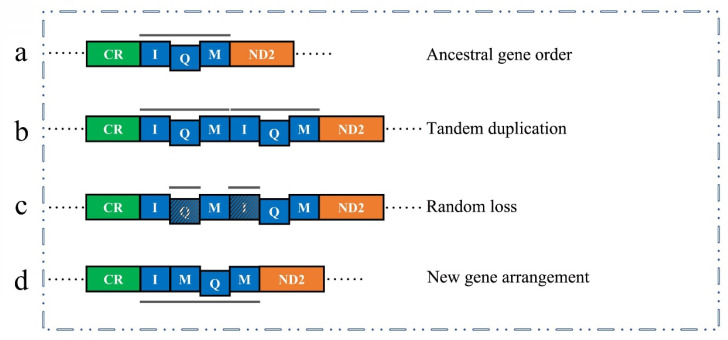
Inferred intermediate processes of the gene rearrangement of Heptageniidae. (**a**) The ancestral gene order of insects. (**b**) Duplication of *trnI*-*trnQ*-*trnM* region. (**c**) Random deletion of the copied genes. (**d**) The final gene order of Heptageniidae.

Compared to the ancestral gene order (Figure 6a), the additional *trnM* was inserted between *trnI* and *trnQ* forming the gene block (*trnI*-*trnM*-*trnQ*-*trnM*-*ND2*). Two models of tandem duplication-random loss (TDRL) and recombination are generally proposed to explain this rearrangement mechanism [53,54]. The hypothetical process of the gene rearrangement is as follows: The gene block (*trnI*-*trnQ*-*trnM*) firstly underwent a tandem duplication, resulting in a new gene cluster with two same sets (*trnI*-*trnQ*-*trnM*-*trnI*-*trnQ*-*trnM*) (Figure 6b). Then this consecutive copy was followed by a random loss; *trnQ* of the first set and *trnI* of the second set were subsequently lost (Figure 6c). Finally, the new gene arrangement of heptageniid mitogenomes (*trnI*-*trnM*-*trnQ*-*trnM*) was generated (Figure 6d). It is plausible to hypothesize that the gene arrangement pattern with an extra *trnM* could be explained through the TDRL model [14,55].

All gene rearrangement events reported in Ephemeroptera are almost focused on the tRNA genes, characterized as minor rearrangement. This has also been found in other insect orders such as Orthoptera, Hemiptera, Hymenoptera [7,56,57]. This minor rearrangement, however, has rarely been used to reconstruct phylogenetic relationships in insects. In our present study, the rearrangement of *trnM* could be considered a clear molecular synapomorphy for Heptageniidae, and might be an effective molecular marker for the family.

### 3.6. Phylogenetic Analyses

Phylogenetic analyses based on PCGs of 16 heptageniid species and 1 siphluriscid species (outgroup) were conducted. Two datasets were used in this study: (1) the amino acid matrix (AA) contained 3661 sites including all PCGs’ aa sequences; (2) the nucleotide matrix (P123) contained 10,983 sites including all PCGs with three codon positions. The phylogenetic trees generated from two analytical methods (BI and ML) had unique topologies with the same dataset.

**Figure 7:**
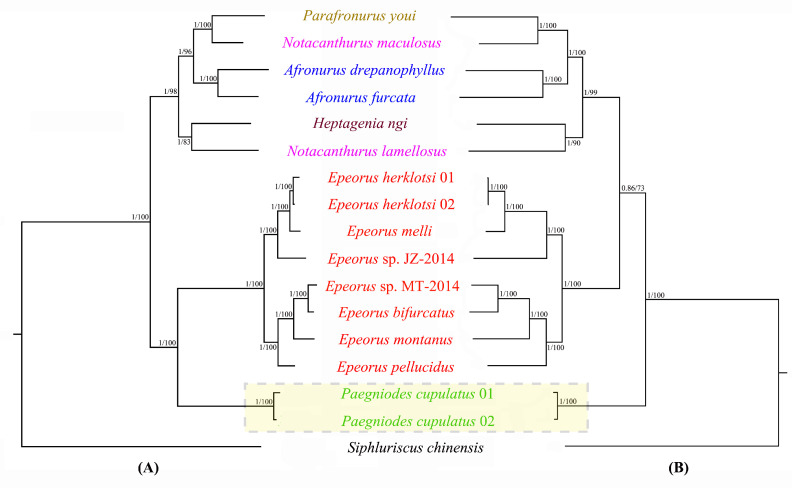
Phylogenetic analyses of Heptageniidae based on amino acid (**A**) and nucleotide datasets (**B**). Numbers separated by a slash on the node are posterior probability (PP) and bootstrap value (BV).

The trees based on two datasets exhibited slightly different topology, the incongruence being restricted to the position of *P. cupulatus* (Figure 7). Monophyly of the genus *Epeorus* received strong support in all analyses, which was consistent with the traditional morphological classification [17]. The AA analysis showed that *P. cupulatus* was the sister group to the species of the genus *Epeorus* (Figure 7A). The results supported the previous analysis that the two genera belong to a monophyletic Rhithrogeninae [17]. However, the P123 tree placed *P. cupulatus* at the base of the clade consisting of all other heptageniid species (Figure 7B). The absence of the *trnM* duplication of *P. cupulatus* seems to support this phylogenetic relationship. Additionally, our previous study found that the genus *Paegniodes* possessed more plesiomorphies in Heptageniidae, such as terminal filament and cerci well-developed, setae scattered on the ventral surface of the maxillae, and gills not enlarged or not forming a friction disk [58]. On the basis of the above analyses, *P. cupulatus* might be recognized as the earliest diverging species among the analyzed heptageniids.

For other species, the positions were identical in all topologies (Figure 7). *Heptagenia ngi* (Heptageniinae) was clustered together with other Ecdyonurinae species, and sister to *N. lamellosus*. Because only one species was sampled in Heptageniinae, its current position within Ecdyonurinae needs expanded samplings to reconfirm in the further study. Our analyses revealed that two species of the genus *Notacanthurus* were not placed together. This is consistent with morphological study, which suggests that the genus is probably not monophyletic, but more likely polyphyletic [59].

## 4. Conclusions

The present study determined ten complete mitogenomes of heptageniid species and is the first detailed comparative genomic and phylogenetic analysis within Heptageniidae. All heptageniid mitogenomes possessed an extra *trnM* except *P. cupulatus,* retaining the typical gene content. The analysis of evolutionary patterns showed that all PCGs were under purifying selection, and that *ND6* and *ND2* exhibited a faster evolution rate and diversity than other genes. A conserved intergenic spacer was observed in all heptageniid mitogenomes, which could be folded into a stable stem–loop structure. A tRNA gene rearrangement was found in most mitogenomes of Heptageniidae and the unique gene block *trnI*-*trnM*-*trnQ*-*trnM*-*ND2* presented. It was plausible to hypothesize that the gene arrangement pattern with an extra *trnM* occurred via the TDRL model. The phylogenetic analyses inferred from mitogenomes strongly supported the monophyly of the genus *Eperous*. Our results improve the understanding of mitogenomic evolution and phylogenetic relationships in Heptageniidae.

## Figures and Tables

**Table 1:** The species information used in phylogenetic analysis.

Subfamily	Species	Size (bp)	GenBank #
Rhithrogeninae	*Epeorus herklotsi* 01	15,502	MG870104
	*Epeorus herklotsi* 02	15,499	MH752075
	*Epeorus* sp. JZ-2014	15,338	KJ493406
	*Epeorus* sp. MT-2014	15,456	KM244708
	*Epeorus montanus*	15,472	This study
	*Epeorus melli*	15,490	This study
	*Epeorus bifurcatus*	15,466	This study
	*Epeorus pellucidus*	15,435	This study
	*Paegniodes cupulatus* 01	15,715	HM004123
	*Paegniodes cupulatus* 02	15721	This study
Ecdyonurinae	*Parafronurus youi*	15,481	EU349015
	*Afronurus furcata*	15,334	This study
	*Afronurus drepanophyllus*	15,242	This study
	*Notacanthurus lamellosus*	15,693	This study
	*Notacanthurus maculosus*	15,524	This study
Heptageniinae	*Heptagenia ngi*	15,495	This study
**Outgroup**			
Siphluriscidae	*Siphluriscus chinensis*	16,616	HQ875717

**Table 2:** Base composition and skewness of heptageniid mitogenomes.

Species	A+T (%)					AT-Skew					GC-Skew				
	All	PCGs	tRNAs	rRNAs	CR	All	PCGs	tRNAs	rRNAs	CR	All	PCGs	tRNAs	rRNAs	CR
*Epeorus herklotsi* 01	65.66	65.10	65.74	65.77	74.45	−0.002	−0.196	−0.018	0.007	0.017	−0.245	−0.033	0.131	0.311	−0.086
*Epeorus herklotsi* 02	65.71	65.15	65.87	65.61	74.80	−0.004	−0.197	−0.020	0.007	0.008	−0.243	−0.031	0.140	0.305	−0.082
*Epeorus* sp. JZ-2014	64.60	64.03	64.62	66.03	72.78	−0.002	−0.201	−0.021	0.012	0.042	−0.209	−0.026	0.123	0.270	0.000
*Epeorus* sp. MT-2014	64.07	62.53	65.19	67.18	78.03	−0.010	−0.197	−0.023	0.027	0.076	−0.232	−0.023	0.112	0.292	−0.164
*Epeorus montanus*	64.86	63.52	64.75	67.31	78.89	−0.019	−0.205	−0.019	0.026	0.082	−0.210	−0.021	0.105	0.262	−0.128
*Epeorus melli*	65.73	65.28	65.01	65.70	74.34	−0.004	−0.199	−0.019	0.004	−0.040	−0.245	−0.022	0.127	0.313	−0.084
*Epeorus bifurcatus*	65.01	63.72	65.64	67.28	78.01	−0.006	−0.194	−0.021	0.026	0.082	−0.245	−0.023	0.111	0.281	−0.255
*Epeorus pellucidus*	66.39	65.56	66.78	67.36	76.64	−0.028	−0.194	−0.011	0.041	0.083	−0.215	−0.017	0.115	0.279	−0.036
*Paegniodes cupulatus* 01	65.59	65.54	65.50	66.41	63.15	−0.009	−0.209	−0.007	0.020	0.154	−0.202	−0.033	0.138	0.313	0.099
*Paegniodes cupulatus* 02	64.73	64.62	66.14	66.59	58.70	−0.005	−0.213	−0.018	0.025	0.244	−0.203	−0.036	0.135	0.296	−0.011
*Parafronurus youi*	66.38	67.04	65.38	66.19	57.03	−0.016	−0.199	−0.020	0.017	−0.003	−0.220	−0.018	0.111	0.309	−0.022
*Afronurus furcata*	64.66	64.85	63.23	65.50	61.22	0.008	−0.184	−0.002	−0.004	0.068	−0.214	−0.021	0.102	0.326	0.096
*Afronurus drepanophyllus*	63.68	63.26	64.31	64.67	66.26	−0.021	−0.204	−0.026	0.021	0.063	−0.176	−0.025	0.115	0.299	0.087
*Notacanthurus lamellosus*	66.26	66.51	64.46	66.68	64.95	−0.046	−0.193	−0.023	0.065	−0.072	−0.167	−0.015	0.141	0.249	−0.225
*Notacanthurus maculosus*	66.77	67.03	65.01	67.17	64.46	−0.014	−0.200	−0.030	0.025	0.045	−0.219	−0.010	0.127	0.298	−0.296
*Heptagenia ngi*	64.12	64.03	62.81	63.95	68.78	−0.005	−0.196	−0.022	−0.008	−0.058	−0.177	0.000	0.121	0.275	−0.094

## Data Availability

The data that support the findings of this study are openly available in National Center for Biotechnology Information at https://www.ncbi.nlm.nih.gov/nuccore (accessed on 10 February 2021), reference numbers MW381291−MW381300.

## Supplementary Materials

The following are available online at https://www.mdpi.com/2075-4450/12/2/170/s1, Table S1. Collection information of Heptageniidae species in this study, Table S2. The information of next generation sequencing for ten samples, Table S3. The partition schemes and best-fitting models selected in amino acid dataset, Table S4. The partition schemes and best-fitting models selected in nucleotide dataset, Table S5. Start and stop codons of protein-coding genes in the mitogenomes of heptageniid mitogenomes.
