# Supplementary material for: Comparative Mitogenomic Analysis of Heptageniid Mayflies (Insecta: Ephemeroptera): Conserved Intergenic Spacer and tRNA Gene Duplication
Source: Insects. 2021 Feb 16;12(2):170. doi: 10.3390/insects12020170 (PMC7920270; doi:10.3390/insects12020170)
Supplement: Supplementary file 1 [file insects-12-00170-s001.zip › Supplementary Materials/Table S1.docx]

**Table S1.** Collection information of Heptageniidae species in this study.

| **Species** | **Locality** | **Date** | **Collector** |
| --- | --- | --- | --- |
| *Afronurus furcata* | Lishui, Zhejiang, China | 03, August 2020 | Zhenxing Ma, Xuhongyi Zheng |
| *Afronurus drepanophyllus* | Dali, Yunnan, China | 18, August 2020 | Ran Li |
| *Heptagenia ngi* | Tianmu Mountain, Zhejiang, China | 05, April 2019 | Wei Zhang, Zhenxing Ma |
| *Epeorus montanus* | Nalati, Xinjiang, China | 05, April 2019 | Na Han |
| *Epeorus melli* | Yiyang, Hunan, China | 14, August 2019 | Ran Li, Wei Zhang |
| *Epeorus bifurcatus* | Leigong Mountain, Guizhou, China | 30, July 2019 | Zhenxing Ma, Xuhongyi Zheng |
| *Epeorus pellucidus* | Yichang, Hubei, China | 04, August 2019 | Ran Li, Wei Zhang |
| *Notacanthurus lamellosus* | Zhangjiajie, Hunan, China | 01, August 2019 | Ran Li, Wei Zhang |
| *Notacanthurus maculosus* | Baiyun Mountain, Henan, China | 10, August 2020 | Wei Zhang, Min Zhang |
| *Paegniodes cupulatus* | Lishui, Zhejiang, China | 14, August 2020 | Zhenxing Ma, Xuhongyi Zheng |
