# Supplementary material for: Comparative Mitogenomic Analysis of Heptageniid Mayflies (Insecta: Ephemeroptera): Conserved Intergenic Spacer and tRNA Gene Duplication
Source: Insects. 2021 Feb 16;12(2):170. doi: 10.3390/insects12020170 (PMC7920270; doi:10.3390/insects12020170)
Supplement: Supplementary file 1 [file insects-12-00170-s001.zip › Supplementary Materials/Table S2.docx]

| Sample Name | Read Number | HQ Reads | HQ Reads |
| --- | --- | --- | --- |
| *Afronurus furcata* | 35,703,750 | 34,341,082 | 96.18% |
| *Afronurus drepanophyllus* | 38,251,178 | 36,768,972 | 96.13% |
| *Heptagenia ngi* | 38,046,160 | 34,899,296 | 91.73% |
| *Epeorus montanus* | 42,894,566 | 41,053,042 | 95.71% |
| *Epeorus melli* | 33,368,284 | 31,655,310 | 94.87% |
| *Epeorus bifurcatus* | 32,627,260 | 30,953,888 | 94.87% |
| *Epeorus pellucidus* | 37,584,882 | 35,572,996 | 94.65% |
| *Notacanthurus lamellosus* | 31,851,682 | 30,324,180 | 95.20% |
| *Notacanthurus maculosus* | 33,661,302 | 31,797,962 | 94.46% |
| *Paegniodes cupulatus* | 36,973,344 | 34,457,168 | 93.19% |

**Table S2.** The information of next generation sequencing for ten samples.
