# Supplementary material for: Comparative Mitogenomic Analysis of Heptageniid Mayflies (Insecta: Ephemeroptera): Conserved Intergenic Spacer and tRNA Gene Duplication
Source: Insects. 2021 Feb 16;12(2):170. doi: 10.3390/insects12020170 (PMC7920270; doi:10.3390/insects12020170)
Supplement: Supplementary file 1 [file insects-12-00170-s001.zip › Supplementary Materials/Table S3.docx]

**Table S3.** The partition schemes and best-fitting models selected in amino acid dataset.

|  | **Nucleotide sequence alignment** | |
| --- | --- | --- |
| **Subset** | **Partition name** | **Best model** |
| Partition 1 | COIII, CYTB, ATP6 | MTMAM+I+G |
| Partition 2 | ATP8, ND3, ND2 | MTMAM+I+G |
| Partition 3 | COI | MTMAM+I |
| Partition 4 | COII | MTREV+G |
| Partition 5 | ND4L, ND5, ND4, ND1 | MTREV+I+G |
| Partition 6 | ND6 | MTMAM+I+G |
