# Supplementary material for: Comparative Mitogenomic Analysis of Heptageniid Mayflies (Insecta: Ephemeroptera): Conserved Intergenic Spacer and tRNA Gene Duplication
Source: Insects. 2021 Feb 16;12(2):170. doi: 10.3390/insects12020170 (PMC7920270; doi:10.3390/insects12020170)
Supplement: Supplementary file 1 [file insects-12-00170-s001.zip › Supplementary Materials/Table S4.docx]

**Table S4.** The partition schemes and best-fitting models selected in nucleotide dataset.

|  | **Nucleotide sequence alignment** | |
| --- | --- | --- |
| **Subset** | **Partition name** | **Best model** |
| Partition 1 | COI_pos1, COII_pos1, ATP6_pos1, CYTB_pos1, COIII_pos1 | SYM+I+G |
| Partition 2 | COI_pos2, COII_pos2, ATP6_pos2, CYTB_pos2, COIII_pos2 | F81+I |
| Partition 3 | COI_pos3, ND2_pos3, ND6_pos3, ATP6_pos3, ND3_pos3, CYTB_pos3, COII_pos3, ATP8_pos3, COIII_pos3 | GTR+I+G |
| Partition 4 | ATP8_pos1, ND3_pos1, ND2_pos1, ND6_pos1 | GTR+I+G |
| Partition 5 | ATP8_pos2, ND2_pos2, ND6_pos2 | GTR+I+G |
| Partition 6 | ND4L_pos1, ND5_pos1, ND4_pos1, ND1_pos1 | GTR+I+G |
| Partition 7 | ND4L_pos2, ND1_pos2, ND3_pos2, ND5_pos2, ND4_pos2 | GTR+I+G |
| Partition 8 | ND1_pos3, ND4L_pos3, ND4_pos3, ND5_pos3 | GTR+I+G |
