# Supplementary material for: Comparative Mitogenomic Analysis of Heptageniid Mayflies (Insecta: Ephemeroptera): Conserved Intergenic Spacer and tRNA Gene Duplication
Source: Insects. 2021 Feb 16;12(2):170. doi: 10.3390/insects12020170 (PMC7920270; doi:10.3390/insects12020170)
Supplement: Supplementary file 1 [file insects-12-00170-s001.zip › Supplementary Materials/Table S5.docx]

| **Species** | **Gene** | | | | | | | | | | | | |
| --- | --- | --- | --- | --- | --- | --- | --- | --- | --- | --- | --- | --- | --- |
|  | ***ATP6*** | ***ATP8*** | ***COX1*** | ***COX2*** | ***COX3*** | ***CYTB*** | ***ND1*** | ***ND2*** | ***ND3*** | ***ND4*** | ***ND4L*** | ***ND5*** | ***ND6*** |
| *Epeorus herklotsi* 01 | ATA/TAA | ATG/TAA | ATT/TAA | ATG/T* | ATG/TAA | ATG/TAG | ATG/TAA | ATG/TAA | ATG/TAG | ATG/T* | ATG/TAA | GTG/T* | ATT/TAA |
| *Epeorus herklotsi* 02 | ATA/TAA | ATG/TAA | ATT/TAA | ATG/T* | ATG/TAA | ATG/TAG | ATG/TAA | ATG/TAA | ATG/TAG | ATG/T* | ATG/TAA | GTG/T* | ATT/TAA |
| *Epeorus* sp. JZ-2014 | ATA/TAA | GTG/TAA | ATT/TAA | ATG/T* | ATG/TAA | ATG/TAG | ATG/TAA | ATG/TAA | ATG/TAG | ATG/T* | ATG/TAA | GTG/T* | ATT/TAA |
| *Epeorus* sp. MT-2014 | ATA/TAA | GTG/TAA | ATT/TAA | ATG/T* | ATG/TAA | ATG/TAG | ATG/TAA | ATG/TAA | ATT/TAG | ATG/T* | ATG/TAA | GTG/T* | ATT/TAA |
| *Epeorus montanus* | ATA/TAA | ATG/TAA | ATT/TAA | ATG/T* | ATG/TAA | ATG/TAG | ATG/TAA | ATG/TAA | ATT/TAG | ATG/T* | ATG/TAA | GTG/T* | ATT/TAA |
| *Epeorus melli* | ATA/TAA | ATG/TAA | ATT/TAA | ATG/T* | ATG/TAA | ATG/TAG | ATG/TAA | ATG/TAA | ATG/TAG | ATG/T* | ATG/TAA | GTG/T* | ATT/TAA |
| *Epeorus bifurcatus* | ATA/TAA | ATG/TAA | ATT/TAA | ATG/T* | ATG/TAA | ATG/TAG | ATG/TAA | ATG/TAA | ATT/TAG | ATG/T* | ATG/TAA | GTG/T* | ATT/TAA |
| *Epeorus pellucidus* | ATA/TAA | ATG/TAA | ATT/TAA | ATG/T* | ATG/TAA | ATG/TAG | ATG/TAA | ATG/TAA | ATT/TAG | ATG/T* | ATG/TAA | GTG/T* | ATT/TAA |
| *Paegniodes cupulatus* 01 | ATA/TAA | ATG/TAA | ACC/TAA | ATG/T* | ATG/TAA | ATG/TAG | ATG/TAA | ATG/TAA | ATC/TAG | ATG/T* | ATG/TAA | ATG/T* | **TTA**/TAA |
| *Paegniodes cupulatus* 02 | ATA/TAA | ATG/TAA | ACC/TAA | ATG/T* | ATG/TAA | ATG/TAG | ATG/TAA | ATG/TAA | ATC/TAG | ATG/T* | ATG/TAA | ATG/T* | TTA/TAA |
| *Parafronurus* youi | ATA/TAA | ATG/TAA | ACC/TAA | ATG/T* | ATG/TAA | ATG/TAG | ATG/TAA | GTG/TAA | ATC/TAG | ATG/T* | ATG/TAA | ATG/T* | ATT/TAA |
| *Heptagenia ngi* | ATA/TAA | GTG/TAA | **ATC**/TAA | ATG/T* | ATG/TAA | ATG/TAG | ATG/TAA | GTG/TAA | ATT/TAG | ATG/T* | ATG/TAA | GTG/T* | ATT/TAA |
| *Afronurus furcata* | ATA/TAA | GTG/TAA | ACC/TAA | ATG/T* | ATG/TAA | ATG/TAG | ATG/TAA | GTG/TAA | ATT/TAG | ATG/T* | ATG/TAA | GTG/T* | ATT/TAA |
| *Afronurus drepanophyllus* | ATA/TAA | GTG/TAA | ACC/TAA | ATG/T* | ATG/TAA | ATG/**TAA** | ATG/TAA | GTG/TAA | ATT/TAG | ATG/T* | ATG/TAA | GTG/T* | ATT/TAA |
| *Notacanthurus lamellosus* | ATA/TAA | GTG/TAA | ACC/TAA | ATG/T* | ATG/TAA | ATG/TAG | ATG/TAA | GTG/TAA | ATT/TAG | ATG/T* | ATG/TAA | GTG/T* | ATT/TAA |
| *Notacanthurus maculosus* | ATA/TAA | GTG/TAA | ACC/TAA | ATG/T* | ATG/TAA | ATG/**TAA** | ATG/TAA | GTG/TAA | ATT/TAG | ATG/T* | ATG/TAA | GTG/T* | ATT/TAA |

**Table S5.** Start and stop codons of protein-coding genes in the mitogenomes of heptageniid mitogenomes.
